# Effects of different induction methods and post-activation potentiation on lower limb muscle activation and explosive power

**DOI:** 10.3389/fbioe.2025.1674571

**Published:** 2025-09-30

**Authors:** Xingchen Zhang, Yuan Gao, Yang Sun, Enjing Li

**Affiliations:** ^1^ School of Physical Education, Yanshan University, Qinhuangdao, Hebei, China; ^2^ Key Lab of Intelligent Rehabilitation and Neuroregulation in Hebei Province, Yanshan University, Qinhuangdao, Hebei, China; ^3^ School of Physical Education and Sports, Central China Normal University, Wuhan, Hubei, China

**Keywords:** blood flow restriction therapy, post-activation potentiation, muscle strength, lower extremity, muscle activation, time factors

## Abstract

**Objective:**

To investigate the acute effects of a different-intensity resistance warm-up on lower limb isokinetic strength, muscle activation, and exercise performance under blood flow restriction.

**Methods:**

Using an isokinetic dynamometer, surface electromyography (sEMG) system, and force platform, lower limb isokinetic strength characteristics, electromyographic parameters, jump kinetics, kinematics, and other relevant parameters were assessed in 15 healthy males following different warm-up induction protocols.

**Results:**

Isokinetic strength testing:HBFR produced higher knee extension torque than LLRT at 3,6,12 min (*P* = 0.012, *P* = 0.028, *P* = 0.019) and surpassed LBFR at 9 min (*P* = 0.015). LBFR increased torque immediately post-warm-up (0 min vs pre: *P* = 0.049), while HBFR peaked at 3 min (*P* = 0.040). Jump performance: HBFR achieved greater flight height than LBFR (*P* = 0.002). At 6 min, LLRT showed lower peak power vs LBFR/HBFR (*P* = 0.046, *P* = 0.034). LBFR increased flight height at 3/6 min (*P* = 0.049, *P* = 0.045), HBFR at 0/3 min (*P* = 0.048, *P* = 0.020). EMG data: LBFR exhibited higher vastus lateralis RMS than HLRT at 9 min (*P* = 0.035). MPF differed significantly between groups across timepoints (*P* = 0.031, *P* = 0.026, *P* = 0.000, *P* = 0.047). HBFR increased vastus medialis RMS at 6 min (*P* = 0.032), while HLRT decreased MPF at 6/12 min (*P* = 0.019, *P* = 0.045).

**Conclusion:**

HBFR warm-up amplifies regional ischemia by superimposing intrinsic and extrinsic constraints, synergistically enhancing neuromuscular recruitment and metabolic stress. This mechanism sustains elevated force output and potentiates PAP, albeit with elevated load-associated injury risks. LBFR warm-up achieves muscle activation comparable to high-intensity training under reduced mechanical loading. The temporal manifestation of PAP exhibits task-specific variability across performance metrics, necessitating individualized BFR protocol optimization and precise recovery time modulation based on target outcomes. Collectively, LBFR represents an efficacious warm-up strategy with minimized injury risk, as evidenced by the present findings.

## 1 Introduction

Lower limb explosive power refers to the ability of the lower limb muscles to generate maximal force in a minimal amount of time (Tanghe and Martin, 2020; Zhang et al., 2021). It serves as a key determinant of performance in various movements such as jumping, sprinting, and change of direction (Cormie et al., 2011), with its biomechanical basis rooted in the contractile dynamics of muscles and the elastic energy storage mechanism of tendons. A range of methodologies, including surface electromyography (sEMG), ultrasound elastography, mechanomyography (MMG), and tensiomyography (TMG), can be employed to evaluate muscle function during such tasks (García-García et al., 2019; Ciobanu et al., 2018; Chiang et al., 2008; Chu and Lee, 2022). Among these, surface electromyography (sEMG), which records the electrical activity associated with muscle contractions, has become the most widely utilized tool due to its non-invasiveness, relative ease of operation, and ability to provide insights into muscle activation, fatigue levels, motor control strategies, and neuromuscular status (Chu and Lee, 2022). Identifying training strategies that can safely and efficiently enhance lower limb explosive power in an acute manner is highly relevant for optimizing athletes’ pre-competition routines and general training programs. Within this context, post-activation potentiation (PAP) is widely recognized as a neurophysiological phenomenon that temporarily improves neuromuscular performance, and it has attracted considerable scientific attention (Zhang et al., 2022). PAP arises from short-term adaptations within the neuromuscular system. The underlying mechanisms may include increased motor neuron excitability, improved efficiency of neural transmission, and phosphorylation of myosin regulatory light chains following high-intensity preconditioning activity. These adaptations enhance contractile dynamics at the muscle fiber level, resulting in a temporary increase in force production and explosive power (Fernández-Galván et al., 2022). Understanding the mechanisms and elicitation strategies of PAP is essential not only for optimizing strength training prescription but also for enhancing athletic performance. Effective induction of potentiation requires preconditioning exercises to exhibit both high intensity and movement pattern specificity. The squat, as a prevalent and efficient multi-joint compound movement, can significantly activate lower limb muscle groups and generate high mechanical load, making it frequently utilized to elicit the PAP effect (Sharma et al., 2018).

To investigate potentiating strategies for back squat preconditioning, this study incorporated blood flow restriction training (BFRT) into the back squat exercise as the primary intervention. BFRT involves applying external pressure using pneumatic cuffs to the proximal portion of the limb during conventional resistance exercise, thereby restricting venous return and partially limiting arterial inflow to the working muscles (Wei et al., 2019; Loenneke et al., 2011; Abe et al., 2006). This approach not only induces a distinct metabolic environment within the muscle but may also affect excitation–contraction coupling and acute load responses by modifying the intrinsic mechanical conditions of the muscle. Due to its combination of high sub-threshold mechanical stress and low absolute load characteristics, BFRT has become an important intervention method in the fields of rehabilitation medicine and sports training (Saatmann et al., 2021). However, most existing studies have focused either on post-activation potentiation (PAP) modulation through single-mode exercises (e.g., traditional back squats) or on the long-term adaptations in strength and power following BFRT (Dankel et al., 2016). Less attention has been devoted to the acute effects of BFRT—particularly how its combination with different intensities of resistance exercise influences subsequent PAP response and immediate power output (Fujita et al., 2007). This represents a significant gap in the current literature.

Based on this background, the present study aims to investigate the effects of varying-intensity back squats performed under BFR on the subsequent PAP response. Specific outcomes include isokinetic strength, vertical jump performance, and muscle activation patterns assessed via surface electromyography (sEMG). The findings are expected to provide a theoretical basis and practical strategies for optimizing lower limb power enhancement protocols.

## 2 Methods

### 2.1 Participants

The required minimum sample size for this experiment was determined *a priori* using G*Power software, resulting in a minimum of 14 participants. To account for potential data loss or invalid data during the study, a total of 15 healthy male university students were recruited as participants. Age: 19.15 ± 1.24 years, height: 180 ± 0.54 cm, body mass: 72 ± 4.25 kg. Participants were included if they met the following criteria: 1) a minimum of 1 year of resistance training experience involving barbell back squats, with a one-repetition maximum (1RM) ≥ 1.25 times body mass, and absence of any contraindications to exercise; 2) no vigorous lower-body resistance exercise within 48 h prior to experimental sessions; 3) no smoking or caffeine consumption within 3 h prior to testing; and 4) no history of lower-limb joint injuries (open or closed), cardiovascular disease, hernia, or other relevant conditions within the preceding 3 months. This study was approved by the Ethics Committee of Qinhuangdao First Hospital (Approval No.: 2025K-124-01) and conducted in accordance with ethical standards. All participants were fully informed of the experimental procedures, voluntarily agreed to participate, and provided written informed consent.

### 2.2 Test method

#### 2.2.1 Testing equipment

Testing equipment included: an isokinetic dynamometer (IsoMed2000, D&R GmbH, Gewerbering Ost 26, 93155 Hemau, GERMANY), a Kistler force platform (Model 9260AA6, Kistler Instruments, Switzerland), a Delsys wireless surface electromyography system (Model SP-W02, Delsys Inc., United States), an Airbands wireless blood flow restriction system (10 cm width, VALD, Australia), a stopwatch, an Olympic barbell, and barbell plates.

#### 2.2.2 Warm-up induction protocol

A repeated-measures design was employed, with each participant completing four experimental sessions. One week prior to the formal experiment, a one-repetition maximum (1RM) back squat assessment was conducted according to National Strength and Conditioning Association (NSCA) guidelines (Haff et al., 2021). Subsequent testing sessions were performed with 1-week intervals between sessions, commencing the week after the 1RM assessment. Testing was performed using four distinct warm-up protocols: 1) Low-intensity resistance exercise combined with blood flow restriction (LBFR), 2) Low-intensity resistance exercise training (LLRT), 3) High-intensity resistance exercise training (HLRT), and 4) High-intensity resistance exercise combined with blood flow restriction (HBFR). The order in which each participant completed the four experimental conditions was determined by a computer-generated random sequence, ensuring that all possible orders of the four sessions had an equal chance of occurring. Resistance loads were set at 30% 1RM for the LBFR and LLRT groups and at 70% 1RM for the HLRT and HBFR groups. The resistance warm-up protocol consisted of 3 sets of 4 repetitions of barbell back squats, with a 30-second rest interval between sets. This total volume is sufficient to induce meaningful neuromuscular activation and potentiation while minimizing the fatigue typically associated with higher-repetition schemes (Su et al., 2023). Three spotters were provided during all squatting exercises. The Airbands wireless BFR cuff was applied to the proximal third of the thigh. Arterial occlusion pressure (AOP) was determined using the device’s integrated pressure sensor prior to testing. A compression pressure equivalent to 60% of the individual’s AOP was applied at this location. This specific pressure was selected based on previous literature indicating that it effectively induces metabolic stress, while simultaneously maximizing participant comfort and tolerability (Wei et al., 2019; Brumitt et al., 2020). All experiments were conducted in a climate-controlled laboratory. The ambient temperature was maintained at approximately 23 °C ± 2 °C, and the relative humidity was approximately 50% ± 10%. Behavioral tests were performed during the light phase (between 9:00 a.m. and 5:00 p.m.) to minimize potential circadian influences.

#### 2.2.3 Experimental procedures

**FIGURE 1:**
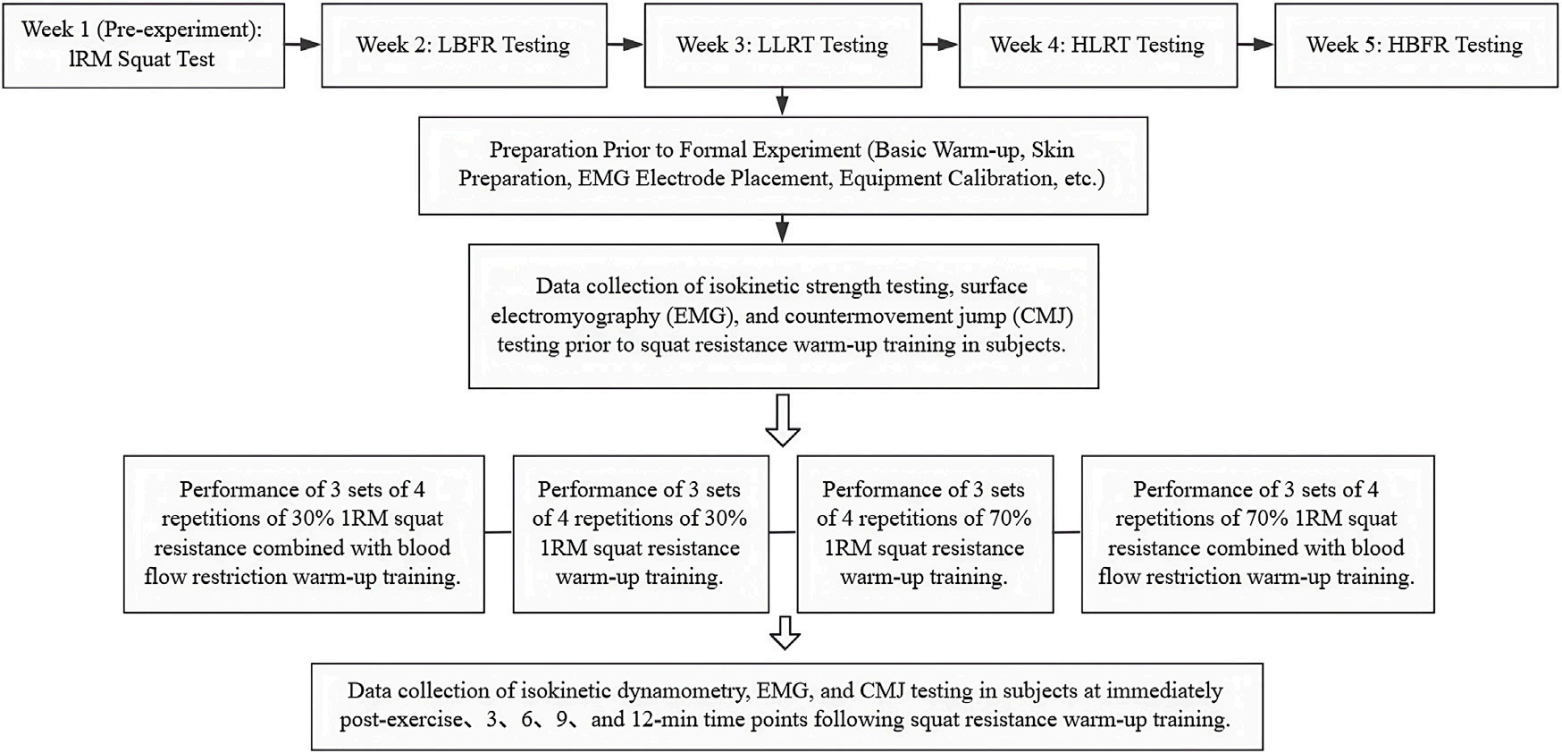
Flow diagram of the experimental protocol.

Upon arrival at the testing facility, participants were registered and provided written informed consent. All participants were informed of the specific protocol conducted during each testing session. They then changed into standardized testing attire and performed 5–10 min of jogging followed by dynamic stretching. Following the baseline warm-up, skin preparation was performed by research staff at the surface electromyography (sEMG) electrode sites. The skin was cleaned with alcohol wipes to remove surface oils, followed by shaving with disposable razors to remove hair. After allowing the alcohol to evaporate, sEMG sensors were applied according to SENIAM guidelines, and signal quality was verified. A pre-warm-up assessment was first conducted. Following a 10-minute rest period, participants performed the warm-up induction protocol, which consisted of 3 sets of 4 repetitions of resistance back squats. Data collection included isokinetic dynamometry, sEMG, and CMJ measurements immediately post-warm-up, and at 3, 6, 9, and 12 min after warm-up completion. Equipment calibration and participant monitoring were maintained throughout all sessions, with data saved in real time. Following testing completion, research staff supervised participants through a structured cool-down and recovery period. The experimental protocol is summarized in Figure 1.

#### 2.2.4 Outcome metrics and measurement procedures

**FIGURE 2:**
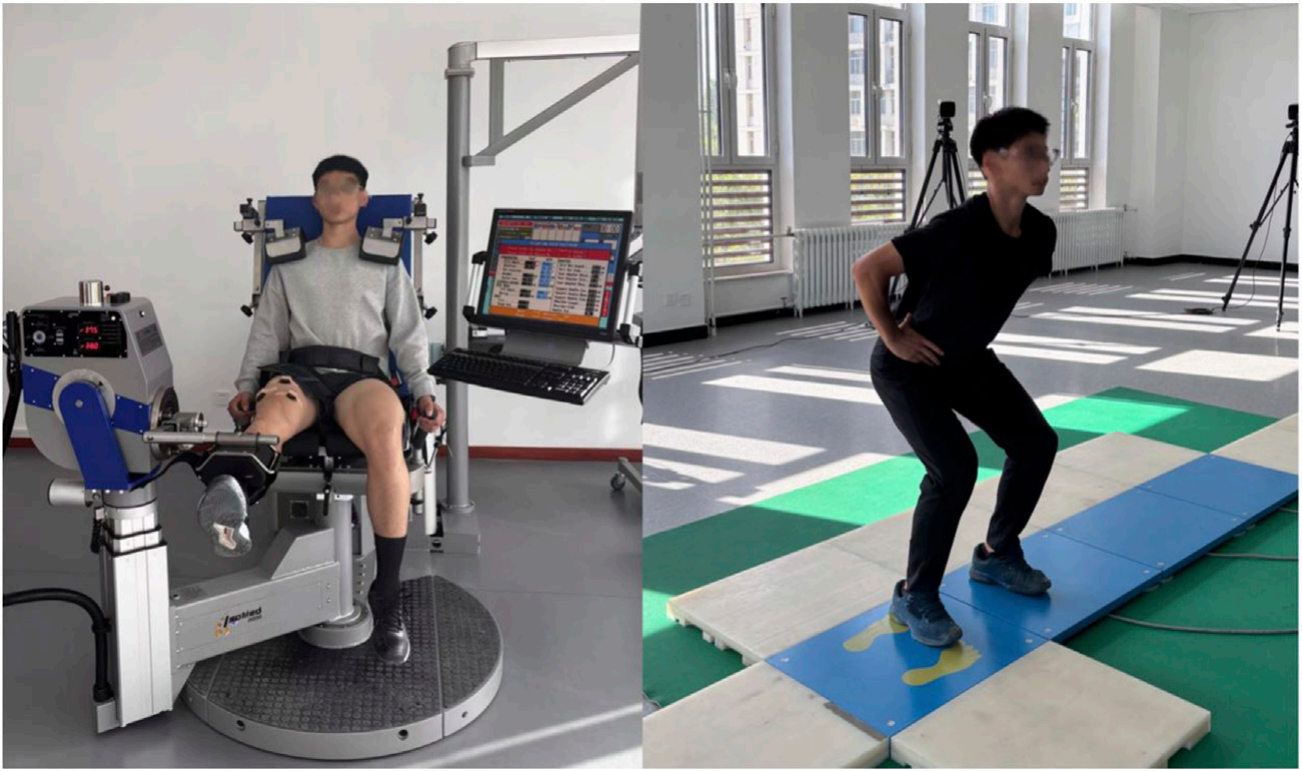
Schematic diagram of isokinetic dynamometry and CMJ testing.

Isokinetic dynamometry quantified dominant-leg knee joint peak torque (limb dominance established through standardized kicking protocol). During testing, subjects maintained seated posture with trunk stabilization, while mechanical restraints immobilized the thigh and ankle segments at predetermined angles. The range of motion (ROM) for the knee joint was individualized for each participant. Testing was performed in seated position using concentric-concentric knee extension-flexion at 180°/s. Participants completed 5 repetitions, with peak torque data from repetitions 2-4 extracted for analysis. Throughout isokinetic testing, synchronized sEMG data were collected from the dominant-leg vastus lateralis (VL), rectus femoris (RF), and vastus medialis (VM) using the Delsys wireless system. Immediately following isokinetic testing, CMJ were performed on a Kistler force platform. Flight height and peak power output during the jump phase were quantified for analysis. During CMJ testing, participants adopted a standardized stance: feet shoulder-width apart with hands fixed on the iliac crests and trunk maintained in vertical alignment. Upon an auditory cue, subjects rapidly descended through combined hip and knee flexion to achieve ∼90° knee angle, followed immediately by maximal vertical propulsion. Two valid trials meeting technical criteria were retained for analysis. EMG signals were processed and analyzed using MATLAB R2019a. Raw data underwent band-pass filtering (4th-order Butterworth, 50-500 Hz cutoff frequencies) (Freitas et al., 2020), followed by full-wave rectification. For each lower-limb muscle during isokinetic testing, the root mean square (RMS) amplitude and median power frequency (MPF) were extracted as primary outcome metrics. EMG signals were normalized in amplitude using the isometric maximum voluntary contraction (MVC). Subjects performed an isometric MVC for the target muscle, and the recorded EMG signal was processed identically to the experimental trials (band-pass filtering, full-wave rectification, and smoothing). The peak value of the resulting envelope was taken as the reference value. Each data point of the envelope signal from the task trials was divided by this peak value and converted to a percentage (%MVC) to obtain normalized amplitude. Three complete effort cycles were selected for feature analysis. The average values of RMS and MPF across the three cycles were computed and used as representative values for subsequent statistical analysis. EMG electrode positions are specified in Table 1. Isokinetic dynamometry testing and CMJ testing procedures are illustrated in Figure 2.

**TABLE 1:** Muscle identification and electrode placement.

Name	Electrode positions
vastus lateralis (VL)	2/3 distally along the line from the anterior superior iliac spine to the lateral patellar border
rectus femoris (RF)	Midpoint of the line between the ASIS and the superior patellar pole
vastus medialis (VM)	4/5 distally along the line from the ASIS to the medial joint space of the knee

### 2.3 Statistical analysis

Data computation and statistical analysis in this study were performed using Excel and SPSS 26.0 software. Data visualization was created with Origin 2021 software. The normality distribution and outliers of the data were first examined. Subsequently, one-way repeated measures analysis of variance (ANOVA) was employed for between-group comparisons to examine differences among different warm-up modalities (LBFR, LLRT, HLRT, HBFR). For the jump test data, isokinetic strength data, and sEMG data, Mauchly’s test of sphericity was conducted. If the result met the Huynh - Feldt condition (*P* > 0.05), the sphericity assumption was accepted, and the results from the univariate ANOVA were used. If the sphericity assumption was violated (*P* ≤ 0.05), the Greenhouse - Geisser correction was applied. Post-hoc multiple comparisons were performed using the Bonferroni correction. Within-group comparisons were conducted using paired-sample t-tests to analyze the effects of recovery time (0, 3, 6, 9, and 12 min) on jump performance, isokinetic strength data, and surface electromyography data. Data are presented as mean ± standard deviation (M ± SD), with statistical significance set at *P* < 0.05.

## 3 Results

### 3.1 Peak torque

**FIGURE 3:**
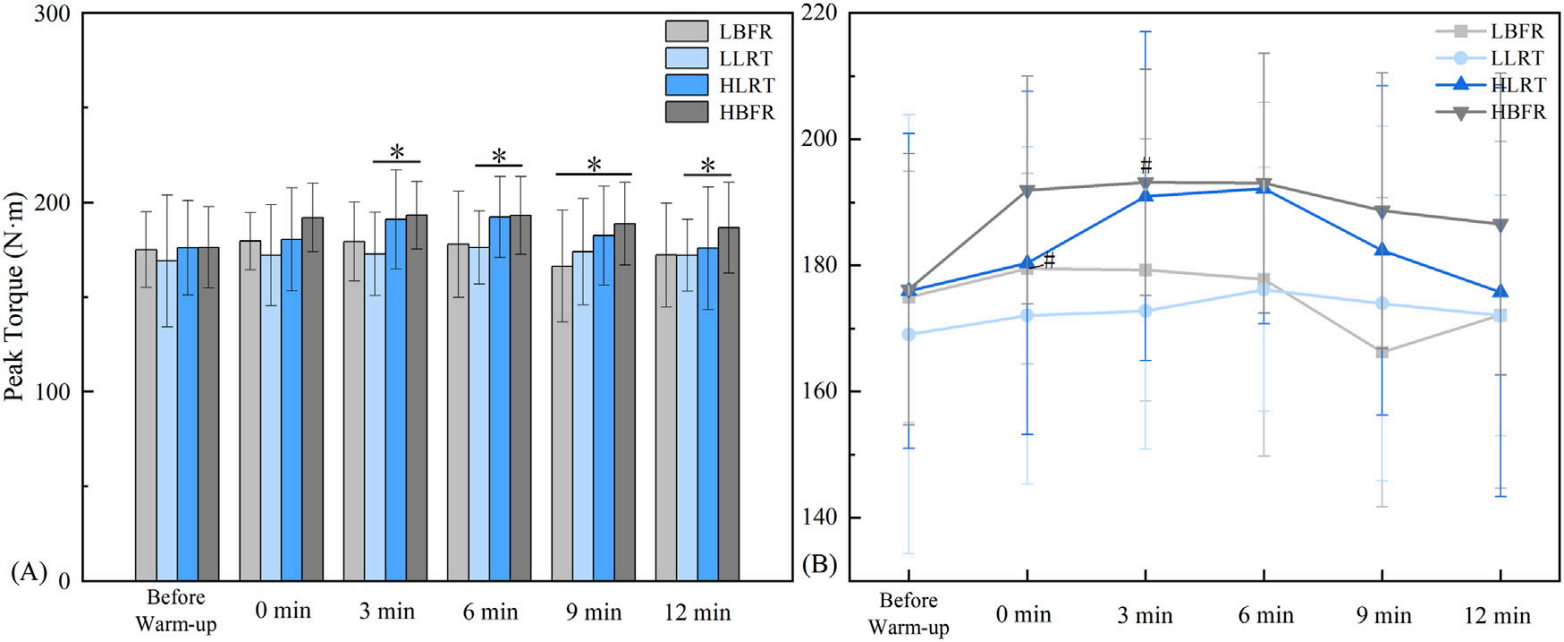
Characteristics of Peak Torque Variation. Note: **(A)** denotes between-group comparisons, * indicates a significant difference at P < 0.05; **(B)** denotes within-group comparisons, # indicates a significant difference compared to Pre-Warm-up. LBFR denotes low-intensity resistance exercise combined with blood flow restriction; LLRT denotes low-intensity resistance exercise training; HLRT denotes high-intensity resistance exercise training; HBFR denotes high-intensity resistance exercise combined with blood flow restriction.

Between-group analysis (Figure 3A) revealed significant differences: at 3 min between LLRT and HBFR groups (*F* = 5.861, *P* = 0.012, *d* = −1.018); at 6 min between LLRT and HBFR groups (*F* = 5.101, *P* = 0.028, *d* = −0.88); at 9 min between LBFR and HBFR groups (*F* = 4.268, *P* = 0.015, *d* = −0.88); and at 12 min between LLRT and HBFR groups (*F* = 2.452, *P* = 0.019, *d* = −0.664). Within-group analysis (Figure 3B) demonstrated significantly greater peak knee extension torque post-warm-up vs pre-warm-up: in the LBFR group at 0 min (*t* = −2.269, *P* = 0.049, *d* = 0.321), and in the HBFR group at 3 min (*t* = −2.405, *P* = 0.040, *d* = 0.423).

### 3.2 Between-group variation characteristics of jump performance

**FIGURE 4:**
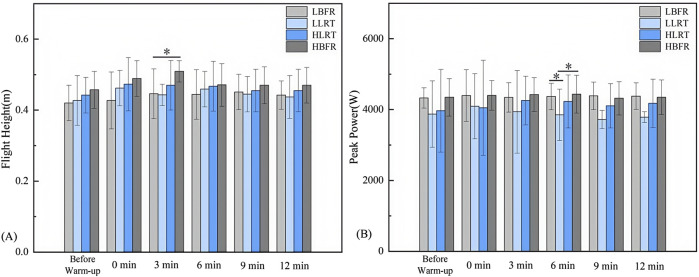
Between-Group Variation Characteristics of Flight Height and Peak Power Output. Note: **(A)** Between-group variation characteristics of CMJ flight height; **(B)** Between-group variation characteristics of CMJ peak power output. * denotes significant difference at *P* < 0.05. LBFR denotes low-intensity resistance exercise combined with blood flow restriction; LLRT denotes low-intensity resistance exercise training; HLRT denotes high-intensity resistance exercise training; HBFR denotes high-intensity resistance exercise combined with blood flow restriction.

Between-group comparisons (Figure 4A) revealed significant differences: at 3 min in flight height between LBFR and HBFR groups (*F* = 4.575, *P* = 0.002, *d* = −1.105); at 6 min in peak power output between LLRT and both LBFR and HBFR groups (*F* = 3.069, *P* = 0.046, *P* = 0.034, *d* = −0.895, *d* = −0.917).

### 3.3 Within-group variation characteristics of jump performance

Within-group analysis (Table 2) demonstrated significantly greater CMJ flight height post-warm-up vs pre-warm-up: in the LBFR group at 3 min (*t* = −2.275, *P* = 0.049, *d* = 0.406) and 6 min (*t* = −2.277, *P* = 0.045, *d* = 0.375); and in the HBFR group immediately post-warm-up (*t* = −2.292, *P* = 0.048, *d* = 0.582) and at 3 min (*t* = −2.834, *P* = 0.020, *d* = 1.13).

**TABLE 2:** Within-group variation characteristics of CMJ flight Height(m).

Recovery time	LBFR	LLRT	HLRT	HBFR
Pre-Warm-up	0.420 ± 0.057	0.427 ± 0.074	0.442 ± 0.056	0.457 ± 0.052
0 min	0.427 ± 0.086	0.462 ± 0.054	0.473 ± 0.075	0.489 ± 0.057*
3 min	0.446 ± 0.071^a^	0.443 ± 0.034	0.470 ± 0.079	0.509 ± 0.038*
6 min	0.444 ± 0.072^a^	0.459 ± 0.058	0.467 ± 0.078	0.471 ± 0.063
9 min	0.451 ± 0.055	0.445 ± 0.059	0.455 ± 0.063	0.470 ± 0.052
12 min	0.442 ± 0.047	0.437 ± 0.060	0.455 ± 0.064	0.470 ± 0.057

^a^ Denotes significant difference versus pre-warm-up at *P* < 0.05. LBFR, denotes low-intensity resistance exercise combined with blood flow restriction; LLRT, denotes low-intensity resistance exercise training; HLRT, denotes high-intensity resistance exercise training; HBFR, denotes high-intensity resistance exercise combined with blood flow restriction.

Within-group analysis (Table 3) revealed no statistically significant differences in CMJ peak power output across recovery time points (*P* > 0.05).

**TABLE 3:** Within-group variation characteristics of CMJ peak power Output(W).

Recovery time	LBFR	LLRT	HLRT	HBFR
Pre-Warm-up	4328.30 ± 285.02	3872.90 ± 935.62	3967.30 ± 1167.61	4346.00 ± 528.66
0 min	4395.10 ± 731.39	4092.70 ± 920.00	4050.40 ± 1342.77	4398.10 ± 417.92
3 min	4342.90 ± 415.91	3937.70 ± 1166.00	4253.90 ± 687.94	4421.00 ± 478.63
6 min	4368.70 ± 375.28	3852.70 ± 724.12	4227.60 ± 747.63	4435.30 ± 532.72
9 min	4385.80 ± 386.35	3717.90 ± 259.14	4104.40 ± 626.46	4316.80 ± 470.60
12 min	4379.70 ± 372.47	3785.90 ± 153.78	4174.20 ± 683.11	4346.30 ± 488.14

### 3.4 Between-group variation characteristics of lower-limb muscle activation

**FIGURE 5:**
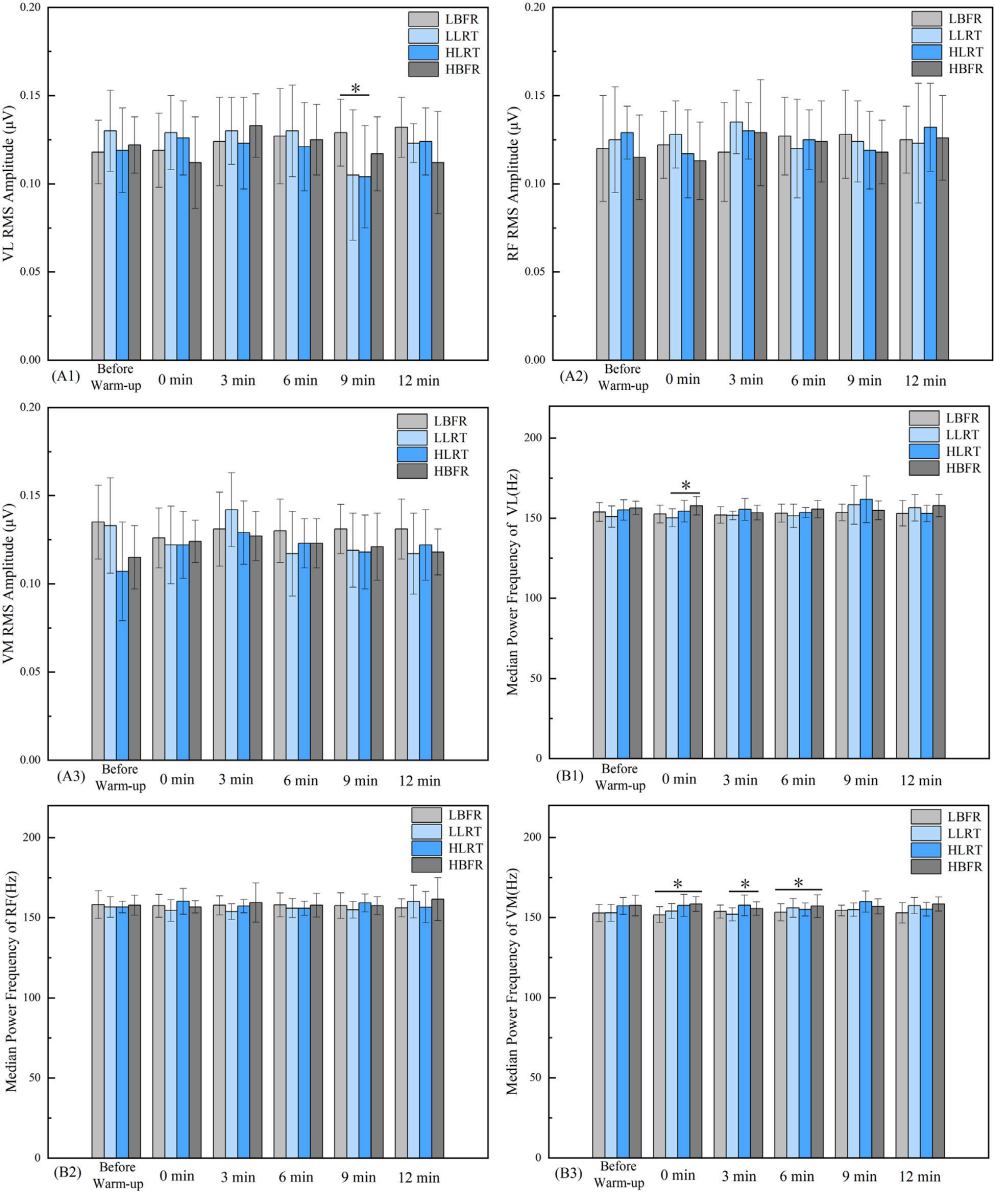
Between-Group Variation Characteristics of Lower-Limb Muscle RMS and MPF. Note: A1 denotes the between-group pattern of change in the RMS value of the vastus lateralis. B1 and B2 indicate the respective patterns of change in the MPF values for the vastus lateralis and rectus femoris muscles. * denotes significant difference between groups at *P* < 0.05. LBFR denotes low-intensity resistance exercise combined with blood flow restriction; LLRT denotes low-intensity resistance exercise training; HLRT denotes high-intensity resistance exercise training; HBFR denotes high-intensity resistance exercise combined with blood flow restriction.

Between-group comparisons revealed a significant difference in vastus lateralis RMS between LBFR and HLRT groups at 9 min (Figure 5A1, *F* = 1.808, *P* = 0.035, *d* = 0.722). At 0 min post-warm-up, significant differences in vastus lateralis MPF were observed between LLRT and HBFR groups (Figure 5B1, *F* = 6.066, *P* = 0.031, *d* = −1.11). At 3 min post-warm-up, vastus medialis MPF differed significantly between LLRT and HBFR groups (Figure 5B3, *F* = 5.447, *P* = 0.026, *d* = −0.843). At 0 min and 6 min post-warm-up, significant differences in vastus medialis MPF emerged between LBFR and HBFR groups (Figure 5B2, *F* = 5.461, *P* = 0.000, *P* = 0.047, *d* = −1.394, *d* = −0.615). No significant differences were found in the RMS and MPF of the rectus femoris (Figure 5A2, 5B2; p > 0.05) or in the RMS of the vastus medialis (Figure 5A3; p > 0.05).

### 3.5 Within-group variation characteristics of lower-limb muscle activation

Within-group analysis (Table 4) demonstrated significantly greater vastus medialis RMS values at 6 min post-warm-up versus pre-warm-up in the HBFR group (*t* = −2.488, *P* = 0.032, *d* = 0.5).

**TABLE 4:** Within-group variation characteristics of RMS values.

Muscle	Time point	LBFR	LLRT	HLRT	HBFR
VM	Pre-Warm-up	0.135 ± 0.021	0.133 ± 0.027	0.107 ± 0.028	0.115 ± 0.018
	0 min	0.126 ± 0.017	0.122 ± 0.022	0.122 ± 0.019	0.124 ± 0.012
	3 min	0.131 ± 0.021	0.142 ± 0.021	0.129 ± 0.028	0.127 ± 0.014
	6 min	0.130 ± 0.018	0.117 ± 0.024	0.123 ± 0.014	0.123 ± 0.014^a^
	9 min	0.131 ± 0.014	0.119 ± 0.021	0.118 ± 0.021	0.121 ± 0.019
	12 min	0.131 ± 0.017	0.117 ± 0.023	0.122 ± 0.020	0.118 ± 0.013

^a^ Denotes significant difference versus pre-warm-up at *P* < 0.05. LBFR, denotes low-intensity resistance exercise combined with blood flow restriction; LLRT, denotes low-intensity resistance exercise training; HLRT, denotes high-intensity resistance exercise training; HBFR, denotes high-intensity resistance exercise combined with blood flow restriction; VM, denotes vastus medialis.

The HLRT group demonstrated significantly lower vastus medialis MPF values at 6 min (*t* = 2.792, *P* = 0.019, *d* = −0.471) and 12 min (*t* = 2.284, *P* = 0.045, *d* = −0.439) post-warm-up compared to pre-warm-up (Table 5).

**TABLE 5:** Within-group variation characteristics of MPF values.

Muscle	Time point	LBFR	LLRT	HLRT	HBFR
VM	Pre-Warm-up	152.820 ± 5.378	152.863 ± 5.334	157.253 ± 5.355	157.476 ± 6.433
	0 min	151.678 ± 5.065	153.999 ± 4.650	157.559 ± 6.971	158.415 ± 4.587
	3 min	153.734 ± 4.041	151.979 ± 4.055	157.614 ± 6.507	155.478 ± 4.244
	6 min	153.208 ± 5.316	155.979 ± 5.872	155.015 ± 4.063^a^	157.094 ± 7.180
	9 min	154.370 ± 3.320	154.892 ± 4.131	159.929 ± 6.689	156.955 ± 4.724
	12 min	152.923 ± 6.365	157.449 ± 5.111	155.134 ± 4.243*	158.372 ± 4.407

^a^ Denotes significant difference versus pre-warm-up at *P* < 0.05. LBFR, denotes low-intensity resistance exercise combined with blood flow restriction; LLRT, denotes low-intensity resistance exercise training; HLRT, denotes high-intensity resistance exercise training; HBFR, denotes high-intensity resistance exercise combined with blood flow restriction; VM, denotes vastus medialis.

## 4 Discussion

The most significant finding of this study is that HBFR warm-up most effectively enhanced peak torque and explosive power, with its superior, intensity-dependent effects emerging after longer recovery due to combined metabolic stress and neural potentiation. Peak torque, the gold standard in isokinetic testing, reflects the maximum force capacity of muscle groups (Wang and Zhou, 2010). The test velocity of 180°/s used here depends on both neural drive and activation of type II muscle fibers (Liu et al., 2025). Results showed that HBFR warm-up produced greater peak knee extension torque than LLRT at multiple recovery time points, and significantly outperformed LBFR during mid-recovery. Further analysis suggests that the acute enhancing effect of blood flow restriction training is intensity-dependent. When high-intensity resistance exercise is combined with BFR, the substantial external load increases mechanical stress and muscular tension. This tension creates endogenous BFR, whose magnitude correlates positively with external load. Consequently, HBFR induces stronger blood flow restriction than other warm-up protocols (Teixeira et al., 2018). When high-intensity resistance exercise is combined with BFR, the substantial external load imposes increased mechanical stress on the muscles, resulting in higher muscular tension. This tension effectively creates endogenous BFR, the degree of which is positively correlated with the external load. As a result, HBFR induces higher levels of blood flow restriction than other warm-up protocols (Teixeira et al., 2018). Additionally, under ischemic conditions, muscle fiber recruitment patterns are believed to be significantly altered. Evidence from previous studies suggests that under ischemic conditions, the recruitment of type II muscle fibers likely surpasses that of type I fibers, departing from the typical sequential low-threshold (type I) to high-threshold (type II) recruitment pattern. The degree of ischemia has been shown to positively correlate with the proportion of type II fibers recruited during exercise (Sundberg, 1994). Consequently, in the HBFR group, the combination of external BFR and intensity-induced endogenous flow restriction may exacerbate local ischemic conditions, thereby potentially enhancing the recruitment of type II fibers (Teixeira et al., 2018). This hypothesis is supported by MPF data from our EMG analysis. An increase in MPF is often interpreted in the literature as being associated with a greater proportional recruitment of type II muscle fibers (Liu and Zhang, 2025); however, it must be acknowledged that spectral shifts can also be influenced by factors such as variations in electrode placement. Given that type II fibers exhibit superior contraction velocity and force generation (Enoka and Duchateau, 2008), this mechanism may explains HBFR’s enhanced peak knee extension torque at multiple recovery time points. These physiological effects were further validated in CMJ outcomes through improved flight height and peak power output.

Between-group analysis indicated that both the LBFR and HBFR groups exhibited significantly higher peak torque after the warm-up compared to baseline, suggesting the presence of PAP, which manifested earlier in the LBFR group. As demonstrated by Suga et al. (2009), low-intensity BFR exercise rapidly induces local ischemia and metabolite accumulation (Suga et al., 2009). These metabolic byproducts potently stimulate group III/IV muscle afferents, which reflexively increase the excitability of spinal α-motoneurons and transiently enhance motor cortex activity (Brandner et al., 2015). Consequently, motor unit recruitment thresholds are lowered and firing rates elevated, leading to improved explosive force production, muscle contraction velocity, and force-generating capacity. Moreover, the relatively low external load in LBFR minimizes structural muscle fatigue, allowing these neural adaptations to translate into immediate gains in peak torque. In contrast, the delayed increase in peak torque observed in the HBFR group until 3 min post-warm-up reflects a more complex recovery process resulting from the combination of high-intensity–induced neuromuscular fatigue and BFR-mediated metabolic stress. The high mechanical load in HBFR inherently contributes to neuromuscular fatigue through both peripheral and central mechanisms (Michaud et al., 2024), while the superimposed BFR further intensifies metabolite accumulation and peripheral fatigue. This combination of high mechanical and metabolic stress is associated with increased markers of muscle damage and delayed-onset muscle soreness compared to traditional training, underscoring the injury risk that necessitates caution (Zhang et al., 2025). Consequently, HBFR required a longer recovery period than LBFR to alleviate peripheral fatigue. However, during CMJ testing, the PAP effect on flight height emerged earlier in the HBFR group than in the LBFR group. This discrepancy can be attributed to fundamental differences in the biomechanical and physiological demands of the two testing modalities. Peak torque measured during isokinetic testing reflects isolated, single-joint maximal voluntary force production, which is highly sensitive to peripheral fatigue induced by metabolites immediately following high-intensity exercise. In contrast, CMJ performance relies on the effective utilization of the stretch-shortening cycle (SSC), tendon elasticity, and neuromuscular coordination—factors that are less compromised by acute metabolic fatigue (Li et al., 2019). The countermovement phase of the CMJ capitalizes on stored elastic energy and stretch reflexes to augment the concentric takeoff, thereby reducing dependence on purely voluntary maximal force generation. This mechanism allows the PAP effect to be expressed earlier despite the presence of residual fatigue. Thus, the earlier appearance of PAP in CMJ compared to isokinetic peak torque may be explained by the task-specific buffering capacity of the neuromuscular system against fatigue. Integrated, multi-joint explosive movements such as the CMJ can benefit more rapidly from enhanced neural drive and increased tendon stiffness, even under conditions of partial fatigue.

Regarding muscle activation, results demonstrated significantly higher vastus lateralis RMS values in LBFR versus HLRT at 9 min recovery. This finding corroborates previous research indicating that low-intensity exercise combined with BFR achieves comparable or even superior muscle activation levels relative to high-intensity resistance warm-ups (Su et al., 2023). Moreover, it demonstrates that low-intensity resistance training with BFR elicits warm-up benefits equivalent to high-intensity protocols through metabolite accumulation and neuromuscular activation mechanisms. Furthermore, studies suggest that blood flow restriction training likely elicits a local hypoxic-ischemic environment, which may reduce muscle fiber recruitment thresholds. This enables low-intensity resistance training to effectively recruit additional fast-twitch fibers, thereby augmenting force-generating capacity (Song et al., 2023). Our findings provide support for this proposed mechanism. Regarding potentiation effects, only the HBFR group exhibited significantly enhanced post-warm-up muscle activation. This likely stems from increased intramuscular lactate concentration and oxygen debt in the occluded limb (Takarada et al., 2000), which necessitates greater fast-twitch fiber recruitment to compensate for impaired energy supply. In contrast, HLRT lacks the metabolic stimulus of blood flow restriction, failing to effectively recruit additional fast-twitch fibers, as potentially indicated by the decrease in median power frequency (MPF) values. Notably, although group-specific temporal windows were observed for these potentiation effects (e.g., some HBFR metrics changed immediately), peak responses predominantly occurred at 3 and 6 min after the warm-up. All groups demonstrated non-monotonic time-dependent patterns in neuromuscular adaptation, marked by an initial decline, followed by augmentation, and a subsequent reduction—a trajectory consistent with previous studies (Kilduff et al., 2007; Jiang et al., 2025; Li et al., 2023). This result further supports the theoretical model proposed by Lowery et al. (2012), in which no clear PAP is detectable immediately post-warm-up due to fatigue-mediated suppression. As recovery proceeds, however, the neuromuscular system gradually overcomes fatigue, allowing potentiation to become increasingly dominant. Accordingly, significant improvements emerge across various performance metrics—including muscular strength, power output, nerve conduction velocity, and motor coordination. This temporal pattern not only reflects the dynamic balance between fatigue and recovery but also underscores the critical importance of tailored warm-up and recovery strategies within athletic performance optimization.

Based on the findings of this study, an optimized warm-up strategy is proposed, emphasizing the need to individualize recovery time according to the specific warm-up protocol and task requirements. For high-frequency, explosive activities performed at a rapid pace, an LBFR warm-up is recommended. This approach rapidly elevates neural excitability through metabolic stimulation, induces relatively little fatigue, and promotes a swift PAP response, thereby requiring a shorter recovery period (0–3 min). In contrast, for tasks requiring single efforts of maximal strength or power output, an HBFR warm-up is more suitable. However, due to the pronounced fatigue induced by high mechanical load combined with ischemic stimulus, the resulting PAP effect is delayed, necessitating a longer recovery duration (3–6 min or more). In practical applications, recovery intervals should be individually monitored and adjusted based on athlete characteristics and the specific demands of the task to maximize performance outcomes.

## 5 Conclusion

This study suggests that the HBFR protocol may amplify regional ischemia by combining intrinsic flow limitation (from high loads) with extrinsic occlusion. This mechanism likely integrates benefits from both neural recruitment and metabolic stress induced by BFR, collectively improving sustained force output and augmenting PAP. While HBFR represents a potent stimulus for enhancing performance, the high external loads raise concerns about potential injury risks, especially under fatigued or competitive conditions. In contrast, LBFR achieves muscle activation levels comparable to HLRT with reduced mechanical effort, primarily through metabolic stimulation under external compression. This lower mechanical strain positions LBFR as a potentially safer alternative for inducing PAP, though its efficacy may depend more heavily on precise occlusion pressure. Additionally, PAP exhibits distinct temporal patterns across testing modalities. Thus, optimizing BFR warm-up protocols—including load intensity, occlusion pressure, and recovery duration—is essential and should be tailored to specific performance objectives. Empirical findings indicate that LBFR may serve as a promising warm-up strategy, enhancing neuromuscular activation and physiological readiness while potentially mitigating risks associated with conventional high-intensity training. Nonetheless, these implications require cautious interpretation, and further research is needed to confirm the efficacy and safety of LBFR under varied conditions.

## 6 Limitations

Furthermore, this study has several limitations. First, the relatively small and homogeneous sample consisting of trained male participants limits the generalizability of the findings. Caution is therefore advised when extrapolating these results to females, untrained individuals, or other populations. Future studies should investigate larger and more diverse cohorts to evaluate potential population-specific differences in responses to combined PAP and blood flow restriction training. Second, although the experimental conditions were administered in randomized order, the repeated-measures design may still be susceptible to order or carryover effects—such as residual fatigue, potentiation, or learning—between sessions. While randomization aimed to distribute these effects evenly across conditions and a standardized rest period was implemented, their potential influence cannot be completely eliminated and should be considered when interpreting within-participant comparisons. Third, although the test movements used in this study are physically demanding and may introduce some degree of interference, this does not compromise the comparisons between warm-up conditions and simultaneously enhances the ecological validity of the experimental design. Finally, the use of a single blood flow restriction pressure and the exclusive focus on acute outcomes preclude any conclusions regarding dose–response relationships or long-term adaptations. Although the acute measures provide valuable insight into the underlying mechanisms, future studies should systematically examine different pressure levels and assess whether these short-term responses lead to sustained functional improvements through longitudinal intervention designs.

## Data Availability

The original contributions presented in the study are included in the article/supplementary material, further inquiries can be directed to the corresponding authors.
